# The Stockholm Pilot study for Lung cancer Screening (Stockholm PLUS): feasibility of baseline low-dose CT lung cancer screening in a high-risk Swedish female population

**DOI:** 10.2340/1651-226X.2026.44826

**Published:** 2026-02-24

**Authors:** Oscar Grundberg, Paulina Kalinowska, Pierre Hillegren, Nicolas Peyrard Janvid, Axel Dimberg, Nina Markholm Nordgren, Fredrik Strand, Vitali Grozman, Gunnar Wagenius

**Affiliations:** a Thoracic Oncology Center, Karolinska University Hospital, Stockholm, Sweden; b Department of Oncology-Pathology, Karolinska Institutet, Stockholm, Sweden; c Department of Radiology, Karolinska University Hospital, Stockholm, Sweden; d Department of Molecular Medicine and Surgery, Karolinska Institutet, Stockholm, Sweden; e Department of Nuclear Medicine and Medical Physics, Karolinska University Hospital, Stockholm, Sweden; f Department of Cardiothoracic Surgery, Karolinska University Hospital, Stockholm, Sweden; g Regional Cancer Centers in Sweden, Stockholm, Sweden

**Keywords:** Lung neoplasm, low-dose computed tomography, early detection of cancer, smoking, artificial intelligence

## Abstract

**Background and purpose:**

Low-dose CT screening of high-risk groups has been shown to reduce lung cancer mortality, and the European Council has therefore recommended that member states explore the feasibility and effectiveness of this approach. In this study, we evaluate the implementation of low-dose CT screening for lung cancer in a Swedish female population.

**Patient/material and methods:**

Women aged 54–74 years in the Southern Stockholm region were contacted via an electronic questionnaire. Individuals who met the same eligibility criteria as in the NELSON trial (a minimum smoking history of 15 pack-years) were invited. The screening consisted of a baseline low-dose computed tomography (LDCT) scan, with the option for a follow-up scan in cases with intermediate findings. Findings were managed in accordance with modified Fleischner Society guidelines.

**Results:**

Between September 2022 and September 2024, 34,580 invitation letters were sent to randomly selected women aged 54–74 years 11,607 individuals (33.4%) completed the questionnaire, whereof 1,106 (10%) met the inclusion criteria. 990 (90%) individuals accepted the invitation and underwent a baseline low-dose CT scan. There were 152 intermediate and 55 positive scans at baseline, and additional eight positive scans at follow-up. Fifteen cases of Lung cancer were found, yielding a positive prediction value (PPV) of 24%. 87% of the lung cancers were in stage IA.

**Interpretation:**

Organized lung cancer screening in a Swedish female population proved feasible, demonstrating a good participation rate, and a cancer detection rate consistent with findings from other major screening trials.

## Introduction

Lung cancer remains the leading cause of cancer-related mortality worldwide [1]. The primary risk factors are age and cumulative exposure to tobacco smoke [2, 3]. In advanced stages, the prognosis is poor and treatment response varies, whereas early-stage lung cancer is curable with surgery or stereotactic body radiotherapy (SBRT) [4], with 5-year survival of 69–82% [5].

Targeted CT screening of high-risk populations – such as heavy smokers – has been an appealing strategy to enable early-stage diagnosis. Early screening studies in the 1900s demonstrated high detection of early-stage lung cancer but were underpowered for mortality [6]. The National Lung Screening Trial (NLST) was the first study to demonstrate a mortality benefit, showing a 20% reduction in lung cancer mortality among heavy smokers (≥ 30 pack-years) screened with low-dose CT (LDCT) compared to chest X-ray [7]. The Nederlands-Leuvens Longkanker Screening Onderzoek trial (NELSON) further established the effectiveness of screening, demonstrating a 24% reduction in 10-year lung cancer mortality in men and a 33% in women, among heavy smokers (≥15 pack-years), compared to an unscreened control group [8, 9]. Although these landmark trials support lung cancer screening, optimal program design remains debated, including eligibility, screening intervals, and nodule management [6].

Since 2014, the U.S. Preventive Services Task Force has recommended annual LDCT screening, with eligibility expanded in 2021 to adults ≥50 years with ≥20 pack-years [10, 11]. Implementation has been slower in Europe despite a 2017 position statement [12]. National screening programs exist in the US, UK, Poland, Croatia, Taiwan, South Korea, and Australia, with Germany preparing to launch and China recommending screening with possible insurance coverage [6, 13–16]. No national program exists in the Nordic countries.

To facilitate implementation, the European Council issued a recommendation in December 2022 urging Member States to ‘explore the feasibility and effectiveness of low-dose CT to screen individuals at high risk for lung cancer, including heavy smokers and ex-smokers, and to link screening with primary and secondary prevention approaches’ [17, 18]. The EU-funded SOLACE project (Strengthening the Screening of Lung Cancer in Europe) supports Member States by providing a personalized implementation toolbox for national and regional centres [13]. Similarly a committee of experts from Nordic Countries have in 2017 recommended to perform CT screening pilot studies in the Nordic countries in order to gain experience and develop specific and safe protocols [19]. Several screening projects have been initiated in Denmark, Norway, and Sweden, and a Finnish randomized screening trial has been published [20].

Due to changing smoking habits, lung cancer incidence in European women has risen steadily, and mortality is projected to surpass breast cancer [21, 22]. According to the 2024 national health survey by the Swedish Public Health Agency, 5.8% (confidence interval [CI]: 5.3–6.3%) of Swedish women were daily smokers. Among 45–64 and 65–84 years old woman, 6.6% (CI 5.7–7.5%) and 7.8% (CI 6.9–8.8%) were daily smokers. In Sweden, women already constitute the majority of lung cancer cases. Yet most screening trials have predominantly enrolled men, leaving some lack of gender-specific data.

The Stockholm Pilot Study for Lung Cancer Screening (Stockholm PLUS), presented here, is the first lung cancer screening study conducted in Sweden. The purpose of this study is to explore feasibility of lung cancer screening in Sweden. Also, to address the lack of gender-specific data, the first part of the study is focused exclusively on women.

## Patients/material and methods

### Study design

This single-center single-arm prospective interventional cohort lung cancer screening study was conducted between September 2022 and September 2024 in Stockholm. It was conducted as a collaboration study between Stockholm City Council (Region Stockholm), the Regional Cancer Center Stockholm-Gotland (RCC SG), and Karolinska University Hospital. The study targeted female participants aged 54–74 years with a smoking history of at least 15 pack-years. Primary objectives included feasibility and reproducibility, with focus on participation rate, detection rates, adverse events, and benign resection rate. A secondary analysis investigated the role of artificial intelligence (AI), focusing on sensitivity/proportion of negative misclassifications, and potential to reduce radiologist workload. The intent of the study was to perform a baseline low-dose-CT (LDCT) on 1,000 high-risk individuals, as well as a 6–12 months’ follow-up for intermediate lung findings. Unlike existing lung cancer screening programs, this pilot study did not include repeated continuous annual or biannual LDCT of participants.

### Ethical considerations

Ethical approval was granted from the National Board for Ethical Review of Sweden, DNR 2021-03356 and 2023-02022-02, and the study was conducted in accordance with the protocol, Good Clinical Practice guidelines, and the Declaration of Helsinki. All participants provided written informed consent.

### Inclusion criteria

The inclusion criteria were based on the NELSON trial. The age limit was, however, adjusted to 55–74 years, which meant that invitations were sent to women who turned 55 during the current year. In practice, this implied that some participants were 54 years old at the time of invitation.

Inclusion criteria were: current daily smoking or cessation within 10 years, smoking history of ≥ 15 cigarettes daily for ≥ 25 years, or ≥10 cigarettes daily for ≥30 years, with minimum 15 pack-years exposure and age 54–74 years.

Exclusion criteria were: smoking cessation ≥ 10 years, ongoing treatment for lung cancer or regular CT examination of the chest.

No consideration was given to obesity, impaired function, or previous cancer in organs other than the lungs.

### Digital infrastructure and recruitment of participants

Study coordination was managed through the Regional Health Control System (RHKS). The coordinating nurse at the Regional Cancer Centre Stockholm-Gotland (RCC SG) sent invitations via RHKS to 34,580 randomly selected individuals. Letters included study information and a QR code to an INCA (Information Network for Cancer Care) web survey with a validated European smoking history questionnaire (Supplementary material). Potential difficulties with the Swedish language or limited access to a computer were not considered. Demographic and smoking characteristics of the invited population, respondents, screening participants, and lung cancer cases are summarized in Table 1. The questionnaire assessed lung cancer history and smoking behaviour (frequency, duration, cessation). RHKS managed participant data and automatically generated CT appointments with electronic referrals for eligible participants, who were offered smoking cessation support via self-referral to the Swedish National Tobacco Quitline. Participation and compliance to smoking cessation were not recorded.

**Table 1:** Demographic and clinical characteristic of randomly invited population, of responding individuals, of the population selected to screening, and of lung cancer cases.

Characteristics	Invited individuals *N* = 34,850	Responding individuals *N* = 11,607	Total screening group invited *N* = 1,106	Lung cancer cases *N* = 15
Age Median [Range]	63 [54–74]	63 [54–74]	62 [54–74]	67 [58–74]
Distribution 54–59 year 60–64 year 65–69 year 70–74 year	10,515/30% 9,740/28% 7,500/21% 6,825/20%	3,603/31% 3,306/28% 2,555/22% 2,143/18%	363/33% 346/31% 249/23% 148/13%	4/27% 3/20% 2/13% 6/40%
Smoking habits Never smoker Current/former smoker*	N.A	5,044/43% 6,563/47%	- 1,106/100%	- 15/100%
Cigarettes/day (among current and former smokers) <10 10–14 >15 Missing data	N.A	397/6% 563/9% 557/8% 5,046/77%	- 556/50% 550/50%	- 5/33% 10/66%
Smoking cessation (among former smokers) <6 month 6 months < 10 years >10 years (excluded from the study and cannot answer the question below)	N.A	120/2% 852/15% 4,661/83%	62/10% 534/90% -	- 6/100% -
Smoking duration <20 years 20–24 years 25–29 years >30 years Missing data	N.A	179/9% 165/9% 284/15% 1,069/56% 205/11%	- 84/8% 195/17% 827/75%	- 2/13% 12/80% 1/7%

* No distinction could be made between former smokers and current smokers.

### Low-dose computed tomography

All examinations were performed on two CT systems: SOMATOM Drive and SOMATOM Confidence (Siemens Healthineers, Forchheim, Germany). Protocols were developed and validated using the N1 Multipurpose Chest Phantom (Kyoto Kagaku Co., Ltd., Japan) to ensure acceptable image quality. The median CTDIvol was 0.36 mGy (IQR 0.15–0.69), and the DLP was 13 mGy·cm (IQR 10–16), corresponding to an effective dose of 0.3 mSv. Dose estimation was performed with ImpactDose v2.3 (CT Imaging GmbH, Erlangen, Germany), which applies pre-calculated Monte Carlo conversion factors. All examinations were performed at Karolinska University Hospital, Stockholm.

### CT reading and nodule management

Nodule management followed locally adapted Fleischner criteria, using the same thresholds as the 2017 guidelines but restricted to maximum diameter, with all nodules assessed uniformly regardless of density. Measurements were performed in multiplanar reconstruction on thin slices (≤1 mm).

All LDCT scans were independently read by two board-certified radiologists (each with > 5 years of experience, subspecialized in lung cancer). In parallel, all images were analyzed by the AI-Rad Companion Chest CT (Siemens Healthineers, Forchheim, Germany) for automated detection and measurement (volume and diameter) of solid nodules. The AI report was available during both readings.

A structured reporting system was developed to categorize participants into five groups (codes 1–5) based on findings (Table 2), each linked to a distinct management pathway. Negative classification (code 1) included no findings, previously investigated benign lesions, clearly benign appearances (e.g. calcification, infection, atelectasis, fibrosis, flat nodules), or nonflat nodules <6 mm; no follow-up was required. Intermediate classification (code 2) was defined as nonflat nodules ≥ 6 mm but <8 mm, followed by repeat LDCT in 6–12 months. Positive classification (code 5) was defined as a nonflat nodule of any attenuation with a maximum diameter ≥ 8 mm, prompting referral for diagnostic work-up at the Thoracic Oncology Center, Karolinska University Hospital. After the 6- to 12-month follow-up LDCT, code 2 cases were reclassified as either code 3 (no further suspicion of malignancy/end of follow-up), code 5 (referral for diagnostic work-up), or code 4 (nodule < 8 mm where malignancy could not be excluded). The latter were no longer followed within the study but were referred for continued follow-up at Karolinska University Hospital, according to clinical routine.

**Table 2:** Classification according to findings on low-dose computed tomography (LDCT).

Classification	Findings	Measure
Code 1 – Negative	No findings Benign findings Nodules < 6 mm	No follow-up
Code 2 – Intermediate	Nodule of any attenuation ≥6 but < 8 mm	LCDT after 6–12 months
Code 3 – End of follow-up	Follow-up LDCT with no further suspicion of malignancy	No follow-up
Code 4 – Further follow-up	Follow-up LDCT showing reference nodule < 8 mm, where malignant or premalignant state cannot be ruled out	Referral for further follow-up
Code 5 – Positive	Nodule of any attenuation ≥ 8 mm	Referral for diagnostic work-up

### Lung cancer cases

Lung cancer cases were defined as individuals with positive screening CT findings (code 5) diagnosed with lung cancer until April 1, 2025. Data on histology, location, stage (IASLC TNM, 9th edition), radiological features, treatment, and molecular alterations were collected. Pulmonary carcinoids, although more indolent, were included in line with NLST and NELSON definitions.

### Artificial intelligence

Ground truth for comparison with AI findings followed the classification system described above, except that prior benign diagnoses were disregarded. For example, a previously known benign 7-mm nodule was assigned code 2 based on size and appearance, rather than code 1.

The AI system output included lung nodule identification, volume, maximum, and mean axial diameters, as well as the maximum 3D diameter. Since the study’s triage criteria were based on maximum diameter, only the maximum 3D diameters were used for performance evaluation of the AI system.

Negative AI misclassification was defined as failure to detect a positive (code 5) or intermediate (code 2) nodule or underestimation of such nodules leading to assignment to a lower risk category. To investigate which types of nodules the AI system misclassified, these nodules were subsequently reclassified according to the ESTI 2025 volume-based cut-offs [19–22]. The proportion of negative classifications corresponds to the inverse of sensitivity.

Workload reduction was defined as the proportion of cases correctly classified as negative based on AI measurement. No other AI performance metrics were considered meaningful in this study, since neither pathological verification, long-term follow-up, nor established volume-based criteria were used as ground truth.

### Management of incidental findings

LDCT reports included statements on lung nodules, as well as significant incidental findings such as severe emphysema or interstitial lung disease. Extrapulmonary findings (e.g. mediastinal lymphadenopathy, severe coronary calcification, osteoporosis, or other tumors) were also documented, with clinically significant findings prompting referral for diagnostic work-up. This report describes lung nodules only while including all adverse events related to work-up of incidental findings.

### Evaluation of screening-related adverse events

Screening-related adverse events were defined according to Common Terminology for Adverse Events (CTCAE) version 4.0. All grade ≥3 adverse events related to LDCT and subsequent screening-related procedures were recorded, including diagnostic CT (± i.v. contrast), PET/CT, bronchoscopy, CT-guided biopsy, surgery, radiotherapy, and systemic therapy.

### Statistical methods and data management

The results in this one-armed study were presented with descriptive statistics. The censor date was set as April 1, 2025. Due to the short observation period, to the noncomparative nature of the study, no survival analyses were performed.

## Results

### Population

Between September 1, 2022, and October 23, 2024, 34,580 invitations were sent to randomly selected women aged 54–74 years in Southern Stockholm. Overall, 11,607 (33.4%) completed the questionnaire; 1,106 (10%) met inclusion criteria and were invited. Of these, 990 underwent baseline LDCT (90% adherence). Demographic and smoking characteristics are summarized in Table 1.

### CT findings and management

At baseline LDCT, 783 (79%) were negative/benign (code 1), 152 (15%) intermediate (code 2), and 55 (6%) positive (code 5). Among intermediate cases, 148 had completed 6–12 month follow-up by April 1, 2025: 99 (67%) were benign (code 3), 41 (28%) continued yearly follow-up (code 4), and eight (5%) became positive (code 5) due to growth to ≥8 mm.

**Figure 1:**
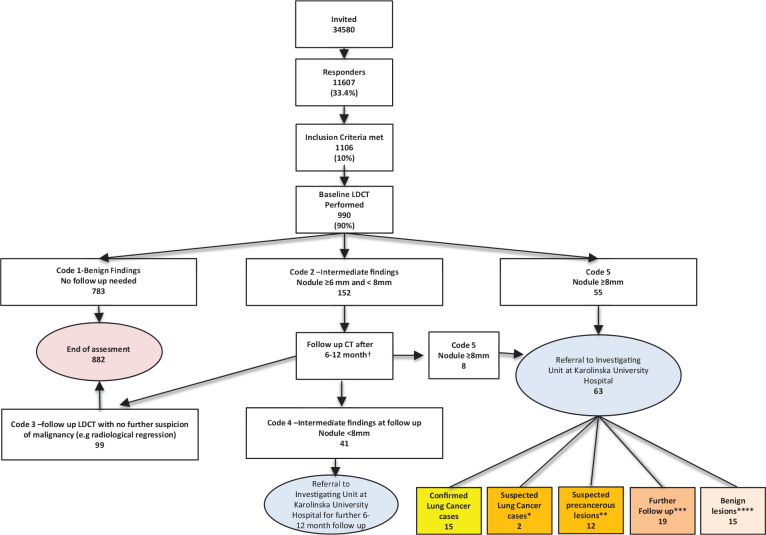
Flowchart of participants in the Stockholm PLUS study, from questionnaire distribution to end of follow-up, diagnosis, or censoring on April 1, 2025.

By April 1, 2025, 63 individuals were classified as positive (code 5) at baseline or follow-up LDCT and referred for diagnostic work-up. Twenty-three underwent invasive procedures: 15 CT-guided fine needle aspiration, four bronchoscopy, and four both. Findings, classification, management, and outcomes are shown in Figure 1 and Tables 3–4. PPV for screen-positive findings before the censor date was 0.24, and the number needed to screen was 66.

*: Two individuals too frail for further investigations or treatments.

**: Cases with subsolid/mostly subsolid lesions are highly suspected of precancerous lesions still under surveillance.

*** : Cases with small lesions with intermediate suspicion of cancer under active surveillance.

****: Two cases of nonmalignant disease were operated; in addition, one case of thoracolithiasis and one biopsied hamartoma were found, and 11 lesions underwent spontaneous regression.

†: At the censoring date, four individuals had not yet performed follow-up CT.

**Table 3:** Screening test results.

Screening uptake (%)	Intermediate findings at baseline (%)	Positive test at baseline (%)	Additional positive test after follow-up of intermediate findings (%)	Total positive tests (%)	Cancer detection	Positive predictive value
1,106/990 (90%)	144/990 (14.5%)	55/990 (5.5%)	8/990 (0.8%)	63/990 (6.3%)	15/990 (1.5%)	0.24

**Table 4:** Lung cancer cases.

Baseline characteristics	*n* (%)
All	15
Age Mean Median (range)	66 67 (58–74)
**Stage** Ia Ib IIa IIb IIIa IIIb IIIc IVa Ivb	13 (87%) 0 0 1 (7%) 0 0 0 1 (7%) 0
**Tumor size in mm** Mean Median (range)	18.4 15 (9–45)
**Histology** Adenocarcinoma Adenosquamous Pulmonary carcinoid	13 (87%) 1 (7%) 1 (7%)
**Radiological appearance** Subsolid Semisolid Solid Cystic	0 9 (60%) 5 (33%) 1 (7%)
**Treatment** Sublobar surgery Lobar surgery SBRT CRT Systemic therapy (chemo-immunotherapy)	10 (67%) 3 (20%) 1 (7%) 0 1 (7%)
**Possible actionable genetic alteration, all** KRAS G12c EGFR exon 21 ALK-mutation VUS	4 (27%) 2 (13%) 1 (7%) 1 (7%)

SBRT: Stereotactic Body Radiation Therapy; CRT: chemoradiotherapy; KRAS: Kirsten Rat Sarcoma; EGFR: Epidermal Growth Factor; ALK: Anaplastic Lymphoma Kinase; VUS: Variant of Unknown significance.

### Lung cancer cases

Fifteen individuals with code 5 lesions were diagnosed with lung cancer: 13 at baseline (1.3%) and 2 at follow-up, yielding a detection rate of 1.5%. Most tumors were stage IA (13/15); one was stage IIB, and all received curative-intent surgery or SBRT. One patient had stage IVa disease and was treated with chemotherapy and checkpoint inhibitors (Table 4). Targetable mutations were identified in 4/15 cases (27%): two KRAS-G12C, one EGFR L858R, and one ALK mutation.

Additionally, 14 code five cases remained undiagnosed/untreated by the censor date, mainly due to small ground-glass lesions. Of the remaining 34 code five cases, 15 were benign and 19 continued radiological follow-up (Figure 1).

### Incidental and false-positive findings

There were two cases in which surgery was performed on non-lung cancer findings. In one case, a CT-guided biopsy raised suspicion of inflammatory lung disease, prompting a diagnostic wedge resection that revealed lymphoid interstitial pneumonia. In another case, CT-guided biopsy and bronchoscopy were inconclusive; the patient subsequently underwent lobectomy, with histopathologic examination showing fibrosis. This final case was classified as a false positive.

### Adverse events

Three cases of grade 3 screening-related adverse events were found, all among individuals diagnosed with lung cancer: one surgery induced atrial fibrillation and one post-op air leakage that required a re-operation. Furthermore, there were one grade 3 immuno-chemotherapy induced episode of neutropenic sepsis, in the patient diagnosed with stage IVa disease.

### Artificial intelligence

All 990 baseline LDCT scans were analyzed with AI-Rad Companion Chest CT (Siemens Healthineers, Forchheim, Germany) for nodule detection and measurement.

The AI failed to detect lesions classified as positive (code 5) or intermediate (code 2) in 23 cases and assigned a lower risk category than the ground truth in an additional six cases. This corresponded to a false-negative rate of 14% and an overall sensitivity of 86%. Importantly, none of the lung cancers treated before the censor date were missed or underclassified by the AI. All 29 discrepant cases were retrospectively reclassified according to the ESTI 2025 criteria and were ultimately considered negative.

The AI classification was true negative in 489 cases, corresponding to a potential 49% reduction in workload, under the same maximum-diameter–based criteria applied in this study.

## Discussion and conclusion

### General discussion

The Stockholm PLUS study is the first lung cancer screening pilot in Sweden. It demonstrated feasibility and reproducibility, with adherence and lung cancer detection rates consistent with other European trials. Overall, these findings support the implementation of lung cancer screening in the Stockholm region.

### Inclusion criteria

Recruitment was based on the Swedish civil registry database. Inclusion criteria were age and smoking history, initially intended to mirror the NELSON trial, which showed improved lung cancer–specific survival in the screening arm. However, the age range was adjusted to 54–74 years following a health economic evaluation [23], in alignment with the NLST trial. Although risk prediction models such as PLCOm2012 [24] are useful for identifying high-risk individuals, they were not applied here due to limited validation at the time of study planning. The results of this study, with an exclusively female population, indicate a feasibility of recruitment and screening process as well as a detection rate consistent with subgroup analyses from both NELSON and the German LUSI trial [9, 25].

### Recruitment and participation

Like several other lung cancer screening trials, the Stockholm PLUS study used a population-based invitation strategy, sending letters to all women in the eligible age range. This contrasts with targeted recruitment in LSUT [26], SUMMIT [27], and YLST [28], which identified smokers through medical records. The population-based method reduces the risk of excluding eligible individuals due to incomplete records. The response rate was 33.4%, similar to NELSON (25%) [9] and the UKLS (30.7%) [29], though lower than recent unpublished Danish trials [30, 31]. Recruitment in NLST [7] and MILD [32] relied on advertising, limiting direct comparison. A socio-economic study will be conducted within the framework of the Stockholm PLUS study, and results will be reported separately.

### Evaluation criteria and findings management

CT findings were classified according to a radiology protocol aligned with Fleischner Society recommendations [33], with local adaptations, ensuring clinical management consistent with local practice, including appropriate follow-up for ground-glass opacities. Volumetric analysis was not included prospectively, as it was not yet recommended by the EU position paper at the time of study planning, and as this study represents a baseline evaluation rather than a full-scale program. Retrospective volumetric analysis will be needed to assess the triage potential of AI. The lack of volumetrics may have influenced management of indeterminate findings. No model-based risk classification (e.g. Brock model [34]) was applied, as these methods require further validation.

### Cancer detection

The lung cancer detection rate of 1.5% was consistent with European trials such as NELSON (0.9%), MILD (0.8%), DANTE (2.2%), ITALUNG (1.5%), DLCST (0.8%), UKLS (1.8%), and LUSI (1.1%), but lower than NLST (3.2%), likely reflecting lower smoking exposure in our cohort.

A high proportion (87%) of cancers were stage IA [25]. This proportion of patients in the early stage exceeded that reported in NELSON (58%), UKLS (67%), MILD (54%), and NLST (50%), suggesting a comparable impact on mortality reduction. Long-term IELCAP data support this, showing 87% 20-year lung cancer–specific survival for stage IA/IB cases [35].

Median age at lung cancer diagnosis was 67 years versus 62 years in the screened cohort, with 56% of cases ≥ 65 years compared to 36% overall, a difference also noted in NELSON. Adenocarcinoma accounted for 87% of cancers, aligning with other European trials. Targetable oncogenic mutations were found in four of 15 cases (27%): two KRAS-G12C, one EGFR L858R, and one rare ALK fusion. While such alterations have limited predictive value in predominantly stage IA NSCLC, they may influence recurrence risk, particularly brain metastases. Molecular profiles of screen-detected cancers have rarely been reported in larger trials.

Comparing PPV across different screening trials is inherently challenging. These difficulties arise primarily from variations in study populations, driven by differing inclusion criteria, as well as differences in radiological evaluation methods and reporting practices. For instance, some trials apply reclassification of findings following follow-up examinations, while others do not. In the two largest randomized trials, reported PPVs varied substantially: 3.6% in the NLST trial and 43.5% in the NELSON trial (male population).

For the Stockholm PLUS study, the most relevant comparison would be with the NELSON trial, given the similar inclusion criteria based on smoking history. However, differences in radiological reporting systems may explain the lower PPV observed in this study. Comparisons with our study are also challenging, as it was based on a single baseline LDCT. The higher PPV reported in the published Finnish study could potentially be explained by the use of the same nodule management protocol as applied in the NELSON trial, namely a volumetric approach with risk stratification based on nodule volume and volume-doubling time [20].

### Benign surgery

In this study, one patient underwent unnecessary surgery for a benign lesion. Among the 16 patients who had curative-intent surgery, this corresponds to a 6% benign resection rate. Although comparisons are limited by the small cohort and short follow-up, the rate was lower than in NLST (23%) and NELSON (28%) and more consistent with I-ELCAP (11%) and more recent studies, such as El Alam et al. (15%) [36].

### Artificial intelligence

Although the AI provided both diameters and volumetric measurements, ground-truth classification was based on maximum diameter (Table 2). Also, according to the criteria of this study, nodules were measured irrespective of density, whereas the AI algorithm was limited to detecting solid nodules (and the solid components of part-solid nodules). This approach is consistent with the ESTI 2025 criteria but resulted in several false-negative classifications in our setting. Importantly, all nodules negatively misclassified by the AI according to the study criteria were ultimately considered negative according to ESTI 2025, suggesting that the true AI sensitivity may be higher than the 86% observed in this study.

Likewise, the observed 49% reduction in radiologist workload, although promising, is likely an underestimate, as workload reduction may be greater when lung findings are assessed using the volume-based approach recommended in the ESTI 2025 criteria.

### Strengths and limitations

The Stockholm PLUS study is the first lung cancer screening study in Sweden, demonstrating feasibility among women with participation, adherence, detection, and benign surgery rates comparable to prior trials. Limitations include its single-round design, small sample size, and absence of volumetric CT assessment, which may have influenced management of intermediate findings.

## Conclusion

The primary objective of this study was to evaluate the feasibility and effectiveness of lung cancer screening in Stockholm. High adherence rates indicate that the program was both efficient and well accepted, while detection rates and the large proportion of early-stage cancers are comparable to NLST and NELSON. Altogether, these findings support the implementation of a structured screening program. A follow-up study will expand the cohort by 2,000 participants, invite the initial cohort for a second screening round, and include men to assess adherence, detection, and generalizability.

## Supplementary Material







## Data Availability

The datasets generated and/or analyzed during the current study are not publicly available due to patient privacy but are available from the corresponding author on reasonable request.
