# Supplementary material for: The Stockholm Pilot study for Lung cancer Screening (Stockholm PLUS): feasibility of baseline low-dose CT lung cancer screening in a high-risk Swedish female population
Source: Acta Oncol. 2026 Feb 24;65:44826. doi: 10.2340/1651-226X.2026.44826 (PMC12946860; doi:10.2340/1651-226X.2026.44826)
Supplement: Supplementary file 1 [file AO-65-44826-s1.pdf]

Supplementary material has been published as submitted. It has not been copyedited, or typeset by Acta Oncologica

Enkätfrågor till undersökningsmålgruppen som är intresserade av att delta i piloten/genomför datortomografi

*Introduktion*

Hej,

Tack för ditt intresse att vara med i pilotstudien kring lungcancerscreening. Med denna enkät kommer studien att välja ut personer som sedan kallas till undersökning med datortomografi. Urvalet av personer görs utifrån studiens fastställda kriterier. Det kan innebära att du inte kommer erbjudas en undersökning med datortomografi.

Enkäten beräknas ta 5 minuter att svara på.

Tack!

XXXX

---

1. Behandlas du för lungcancer eller undersöks du regelbundet över bröstkorgen med datortomografi?
   a. Ja
   b. Nej

2. **Röker du tobak?** *(envalsfråga)*
   a. Ja.
   b. Nej.

3. *Om ja på fråga 2:* **Hur länge har du rökt?** *(envalsfråga)*
   a. Mindre 20 år
   b. 20–24 år
   c. 25–29 år
   d. 30 år eller mer

4. *Om ja på fråga 2*: **Hur många cigaretter per dag har du i genomsnitt rökt?** *(envalsfråga)*
   a. Färre än 10 cigaretter/dag
   b. 10–14 cigaretter/dag
   c. 15 cigaretter/dag eller mer

5. *Om nej på fråga 2:* **Har du tidigare rökt?** *(envalsfråga)*
   a. Ja
   b. Nej

6. *Om ja på fråga 5:* **När slutade du att röka?** *(envalsfråga)*
   a. Inom de senaste 10 åren
   b. För mer än 10 år sedan

7. *Om ja på fråga 5:* **Hur länge rökte du?** *(envalsfråga)*
   a. Mindre 20 år
   b. 20–24 år
   c. 25–29 år
   d. 30 år eller mer

8. *Om ja på fråga 5:* **När du rökte, hur många cigaretter per dag rökte du i genomsnitt?** *(envalsfråga)*
    a. Färre än 10 cigaretter/dag
    b. 10–14 cigaretter/dag
    c. 15 cigaretter/dag eller mer

9. *Om ja på fråga 2:* Har du planer på att sluta röka? *(envalsfråga)*
    a. Ja
    b. Nej
    c. Vet inte

10. *Om a eller c på fråga 9*: Länk till Sluta-Röka-Linjen

11. Stämmer dina svar? Du har nu möjlighet att gå tillbaka i enkäten och titta igenom dem. När du är klar kan du trycka på ”Skicka enkäten”.

*Efter denna fråga finns inte längre möjlighet att gå tillbaka i enkäten.*

12. *Om 15 cigarettes/day during >25 years; or >10 cigarettes/day during >30 years; if former smokers, quitting time ≤10 years:*

Tack för din medverkan. Du är aktuell för lungcancerscreening. Du kommer att få en kallelse till undersökning inom 6–8 veckor. Kontaktuppgifter till Enheten för cancerprevention och screening på RCC Stockholm Gotland.

13. Om **INTE** 15 cigarettes/day during >25 years; or >10 cigarettes/day during >30 years; if former smokers, quitting time ≤10 years:

Tack för din medverkan. Du är inte aktuell för denna studie. Ditt svar är betydelsefullt för det fortsatta arbetet.
