# Supplementary material for: The Stockholm Pilot study for Lung cancer Screening (Stockholm PLUS): feasibility of baseline low-dose CT lung cancer screening in a high-risk Swedish female population
Source: Acta Oncol. 2026 Feb 24;65:44826. doi: 10.2340/1651-226X.2026.44826 (PMC12946860; doi:10.2340/1651-226X.2026.44826)
Supplement: Supplementary file 2 [file AO-65-44826-s2a.pdf]

Supplementary material has been published as submitted. It has not been copyedited, or typeset by Acta Oncologica

Region Stockholm

REGIONALT CANCERCENTRUM RCC STOCKHOLM GOTLAND

KAROLINSKA UNIVERSITETSSJUKHUSET

Regionalt cancercentrum Stockholm Gotland
Enheten för cancerprevention och screening
Box 300 17 LC
104 25 Stockholm

Enkätkod	Personlig kod

Vill du delta i en forskningsstudie gällande lungcancerscreening?

Då lungcancer ökar i Sverige pågår en forskningsstudie gällande införande av lungcancerscreening med syfte att upptäcka eventuell sjukdom i tidigt skede. Forskningsstudien beräknas avslutas under 2026. Bakom studien står Regionalt cancercentrum Stockholm Gotland och Karolinska Universitetssjukhuset.

Cancerscreening är en metod för att hitta tidig och botbar cancer hos individer som inte har några symtom. Resultat från tidigare studie har visat att det finns ett intresse bland kvinnor att få göra regelbunden undersökning med lågdos-datortomografi av lungorna för att upptäcka lungcancer i tidigt skede.

Är du intresserad av att delta?

Första steget för att anmäla sitt intresse att delta i forskningsstudien är att besvara en webbenkät. Att besvara enkäten tar några minuter. Genom att besvara enkäten ger du ditt samtycke till att delta i forskningsstudien.

Utifrån svaren i enkäten görs ett urval av personer som kommer att få en kallelse till röntgenkliniken vid Karolinska Universitetssjukhuset Solna. De som inte erbjuds en undersökning uppfyller inte de kriterier som valts ut för denna studie.

Du når enkäten via webbadress: **svar.incanet.se eller använd QR-koden till höger genom att rikta mobiltelefons kamera mot koden.** Överst i brevet i den gröna rutan finns en enkätkod och en personlig kod som du använder för att logga in till enkäten.

Hur går studien till?

De som tillfrågas att delta i studien är personer 55–74 år. De som uppfyller kriterierna erbjuds att genomgå en lågdos-datortomografi. Besöket tar ungefär 20 minuter. Svaret på undersökningen kommer att skickas till dig när röntgenbilderna analyserats.

Att delta i forskningsstudien är frivilligt och deltagandet kan avbrytas när som helst. Undersökningen är kostnadsfri. De som röker erbjuds rökavvänjningsstöd.

Kontakt

Har du frågor om forskningsstudien gällande lungcancerscreening kontakta Enheten för cancerprevention och screening vid Regionalt cancercentrum Stockholm Gotland

E-post: halsound.hsf@regionstockholm.se
Telefon: 08-123 138 95
Telefontid: måndag–torsdag kl. 10–11

Har du frågor om cancer kontakta Cancerrådgivningen

Telefon: 08-123 138 00
Telefontid: helgfri måndag 8.30–19.30, helgfri tisdag–fredag 8:30–16, övriga tider kan du lämna meddelande.
E-post: cancerradgivningen.hsf@regionstockholm.se

Mer information om att delta i forskningsstudien kan du läsa på baksidan av detta brev. →

Behandling av personuppgifter/sekretess

Att delta i forskningsstudien kommer inte att påverka din fortsatta kontakt med sjukvården. Dina enkät- och provsvar kommer att sparas och endast ansvariga för forskningsstudien har tillgång till informationen. Enligt EU:s dataskyddsförordning har du rätt att kostnadsfritt ta del av de uppgifter om dig som hanteras i studien. Du kan också begära att uppgifter om dig raderas samt att behandlingen av dina personuppgifter begränsas. Om du vill ta del av uppgifterna ska du kontakta huvudansvarig forskare Gunnar Wagenius. Dataskyddsombud hos Hälso- och sjukvårdsförvaltningen nås på 08-123 132 00. Om du är missnöjd med hur dina personuppgifter behandlas har du rätt att inkomma med klagomål till Integritetsskyddsmyndigheten, www.imy.se

Försäkring: Den sedvanliga patientförsäkringen

Frågor och svar om forskningsstudien

Om jag vill delta i studien, är det då ett krav att jag ska sluta röka?
Nej, det är inget krav. Om du vill sluta röka och önskar stöd så är du välkommen att kontakta Sluta-röka-linjen på telefon 020-840 000
Du kan läsa mer på slutarokalinjen.se

Är det bara rökare som drabbas av lungcancer?
Nej, men det är den vanligaste orsaken till lungcancer.

Hur lång tid tar det att få besked om man får delta i forskningsstudien?
Du får svar direkt efter att du har besvarat enkäten om du kommer att få en kallelse till undersökning av lungorna med lågdos datortomografi.

Frågor och svar om undersökning med datortomografi

Hur går undersökningen till?
När du ska undersökas får du ligga på en brits som förs genom den ringformade öppningen på datortomografen som är en röntgenapparat. Det gör inte ont att undersökas men du behöver ligga still. Undersökningen tar endast några minuter och hela besöket ungefär 20 minuter.

Vad är datortomografi?
Det är en särskild form av röntgen som skapar mycket detaljerade bilder av kroppens organ.
På så sätt kan läkare lättare upptäcka sjukdom. Läs mer om datortomografi på 1177.se

Hur lång tid tar det att få besked från undersökningen med datortomografi?
Svaret på undersökningen kommer att skickas till dig när röntgenbilderna analyserats.

Om det visar sig att jag har en förändring i lungan, vad händer då?
– Hittas en mindre förändring i din lunga vid undersökningen kommer du att följas upp med en ny datortomografi efter 6–12 månader. Om förändringen vid uppföljningen inte har ökat i storlek så kommer inga fler uppföljningar att göras.
– Hittas en större förändring kommer du att få tid till specialistklinik vid Karolinska Universitetssjukhuset för vidare utredning.

Kan man hitta förändringar i lungan vid en datortomografi som inte visar sig vara cancer?
Det är vanligt att hitta mindre förändringar vid en datortomografi.
Förändringen kan vara ett ärr eller en inflammation.

Kan man vid undersökningen hitta andra sjukdomar i bröstkorgen?
Ja det kan man. I sådant fall remitteras man till rätt vårdgivare.

Ansvariga för studien

Karolinska Universitetssjukhuset Solna
Gunnar Wagenius, Överläkare, Karolinska Universitetssjukhuset Solna.
E-post: halsound.hsf@regionstockholm.se

Regionalt cancercentrum Stockholm Gotland
Miriam Elfström, verksamhetsutvecklare
E-post: halsound.hsf@regionstockholm.se

Läs om studien på

cancercentrum.se/stockholm-gotland/vara-uppdrag/prevention-och-tidig-upptackt under regionspecifikt innehåll finns mer information. Nå webbsidan direkt med QR-koden nedan genom att rikta din mobiltelefons kamera mot koden.

V20240314
