# Supplementary material for: The Stockholm Pilot study for Lung cancer Screening (Stockholm PLUS): feasibility of baseline low-dose CT lung cancer screening in a high-risk Swedish female population
Source: Acta Oncol. 2026 Feb 24;65:44826. doi: 10.2340/1651-226X.2026.44826 (PMC12946860; doi:10.2340/1651-226X.2026.44826)
Supplement: Supplementary file 3 [file AO-65-44826-s2b.pdf]

Supplementary material has been published as submitted. It has not been copyedited, or typeset by Acta Oncologica

Electronic supplement 2a – English translation

English translation – Electronic supplement 2a

Would you like to participate in a research study on lung cancer screening?
As lung cancer is increasing in Sweden, a research study is ongoing regarding the implementation of lung cancer screening, with the aim of detecting potential disease at an early stage. The research study is expected to be completed in 2026. The study is conducted by the Regional Cancer Centre Stockholm Gotland and Karolinska University Hospital.

Cancer screening is a method used to detect early and potentially curable cancer in individuals who do not have any symptoms. Results from a previous study have shown that there is an interest among women in undergoing regular low-dose CT examinations of the lungs in order to detect lung cancer at an early stage.

Are you interested in participating?
The first step to express interest in participating in the research study is to complete a web-based questionnaire. The questionnaire takes only a few minutes to complete. By completing the questionnaire, you provide your consent to participate in the research study.

Based on the questionnaire answers, a selection of individuals will be invited for an examination at the Radiology Department at Karolinska University Hospital, Solna. Those who are not offered an examination do not meet the criteria selected for this study.

You can access the questionnaire via the website: svar.incanet.se, or by using the QR code on the right by pointing your mobile phone camera at the code.

At the top of this letter, in the green box, you will find a questionnaire code and a personal code, which you use to log in to the questionnaire.

How does the study work?
Those invited to participate are individuals aged 55–74 years. Those who meet the criteria will be offered a low-dose CT scan. The visit takes approximately 20 minutes. The result of the examination will be sent to you once the CT images have been analysed.

Participation in the research study is voluntary and may be discontinued at any time. The examination is free of charge. Individuals who smoke will be offered support for smoking cessation.

Contact
If you have questions about the lung cancer screening research study, please contact the Unit for Cancer Prevention and Screening at the Regional Cancer Centre Stockholm Gotland.

Email: halsound.hsf@regionstockholm.se
Telephone: 08-123 138 95
Telephone hours: Monday–Thursday, 10:00–11:00

If you have questions about cancer, please contact the Cancer Advisory Service:
Telephone: 08-123 138 00
Telephone hours: weekdays Monday 8:30–19:30, Tuesday–Friday 8:30–16:00. At other times you may leave a message.
Email: cancerradgivningen.hsf@regionstockholm.se

More information about participating in the research study can be found on the back of this letter.

Processing of personal data / confidentiality
Participating in the research study will not affect your future contact with healthcare services. Your questionnaire and test results will be stored and only those responsible for the research study will have access to the information.

According to the EU General Data Protection Regulation (GDPR), you have the right to free access to the information about you that is processed in the study. You may also request that your personal data be erased and that the processing of your personal data be restricted. If you wish to access your data, please contact the principal investigator Gunnar Wagenius.

The Data Protection Officer at the Health and Medical Care Administration can be reached at 08-123 132 00.

If you are dissatisfied with how your personal data are being processed, you have the right to submit a complaint to the Swedish Authority for Privacy Protection (Integritetsskyddsmyndigheten), www.imy.se.

Insurance: Standard patient injury insurance.

Questions and answers about the research study

If I want to participate, is it required that I quit smoking?
No, it is not required.
If you want to quit smoking and would like support, you are welcome to contact the Swedish National Tobacco Quitline (Sluta-Röka-Linjen) by telephone: 020-840 000.
You can read more at slutarokalinjen.se.

Is it only smokers who develop lung cancer?
No, but smoking is the most common cause of lung cancer.

How long does it take to receive information about whether I will be able to participate in the research study?
You will receive an answer immediately after you complete the questionnaire as to whether you will receive an invitation for a low-dose CT examination of the lungs.

Questions and answers about CT examinations

How is the examination performed?
During the examination, you will lie on a table that is moved through the ring-shaped opening of the CT scanner, which is an X-ray imaging device. The examination is painless, but you will need to lie still. The scan takes only a few minutes, and the entire visit takes approximately 20 minutes.

What is computed tomography (CT)?
Computed tomography is a specialised form of X-ray imaging that produces highly detailed images of the body’s organs. This helps doctors detect disease more easily. You can read more about CT at 1177.se.

How long does it take to receive the results of the CT examination?
The results of the examination will be sent to you once the CT images have been analysed.

If it turns out that I have a change in the lung, what happens then?
- If a smaller change is found in your lung during the examination, you will be followed up with a new CT scan after 6–12 months. If the change has not increased in size at follow-up, no further follow-up will be performed.
- If a larger change is found, you will receive an appointment at a specialist clinic at Karolinska University Hospital for further evaluation.

Can changes in the lung be found on CT that do not turn out to be cancer?
Yes. It is common to find small changes on CT. The change may represent a scar or inflammation.

Can the examination detect other diseases in the chest?
Yes. In such cases, you will be referred to the appropriate healthcare provider.

Read more about the study
Read more about the study at:
cancercentrum.se/stockholm-gotland/vara-uppdrag/prevention-och-tidig-upptackt
Under “region-specific content” you will find more information.

You can also access the webpage directly using the QR code below by pointing your mobile phone camera at the code.

Responsible for the study
Karolinska University Hospital, Solna
Gunnar Wagenius, Senior Consultant (Attending Physician), Karolinska University Hospital, Solna
Email: halsound.hsf@regionstockholm.se

Regional Cancer Centre Stockholm Gotland
Miriam Elfström, Development Officer
Email: halsound.hsf@regionstockholm.se

Version: V20240314
